# Comparison of Explant-Derived and Enzymatic Digestion-Derived MSCs and the Growth Factors from Wharton's Jelly

**DOI:** 10.1155/2013/428726

**Published:** 2013-04-09

**Authors:** Jong Hyun Yoon, Eun Youn Roh, Sue Shin, Nam Hee Jung, Eun Young Song, Ju Young Chang, Byoung Jae Kim, Hye Won Jeon

**Affiliations:** ^1^Department of Laboratory Medicine, Seoul National University College of Medicine, Seoul 110-744, Republic of Korea; ^2^Department of Laboratory Medicine, Seoul National University Boramae Hospital, 425 Shindaebang-dong, Dongjak-gu, Seoul 156-707, Republic of Korea; ^3^Seoul Metropolitan Public Cord Blood Bank, Allcord, Seoul 156-707, Republic of Korea; ^4^Department of Pediatrics, Seoul National University Boramae Hospital, Seoul 156-707, Republic of Korea; ^5^Department of Obstetrics and Gynecology, Seoul National University Boramae Hospital, Seoul 156-707, Republic of Korea

## Abstract

Wharton's jelly is not only one of the most promising tissue sources for mesenchymal stem cells (MSCs) but also a source of natural growth factors. To prove that we can get both natural growth factors and MSCs from Wharton's jelly, we compared cellular characteristics and the level of basic fibroblast growth factor (bFGF) from samples using the explant culture method to those derived from the traditional enzymatic culture method. The levels of bFGF were 27.0 ± 11.7 ng/g on day 3, 15.6 ± 11.1 ng/g on day 6, and decreased to 2.6 ± 1.2 ng/g on day 14. The total amount of bFGF released was 55.0 ± 25.6 ng/g on explant culture. Compared with the traditional enzymatic digestion method, the explant culture method showed a tendency to release higher levels of bFGF in supernatant media for the first week of culture, and the higher cellular yield at passage 0 (4.89 ± 3.2 × 10^5^/g versus 1.75 ± 2.2 × 10^5^/g, *P* = 0.01). In addition, the genes related to mitosis were upregulated in the explant-derived MSCs.

## 1. Introduction

A variety of functions of mesenchymal stem cells (MSCs) in cellular therapeutics have been studied: homing to target tissue, repairing damaged cells (replacement), stimulating host cell regeneration, autocrine/paracrine/intracrine effects, and immune modulation [1, 2]. Supported by promising preclinical studies [3, 4], many clinical trials have used MSCs from a variety of sources for indications such as central nervous system diseases (e.g., ischemic stroke, Parkinson's disease, and spinal cord injury), heart disease, diabetes mellitus, acute graft versus host disease, and some autoimmune diseases (http://www.clinicaltrials.gov/). Such therapeutic applications require adequate quantities and qualities of MSCs.

Although MSCs can be isolated from almost all tissues, including liver, lung, fetal pancreas, trabecular bone, synovium, skeletal muscle, deciduous teeth, and peripheral blood, the byproducts of birth, such as placenta, amniotic membrane, umbilical cord (UC), and umbilical cord blood (UCB) [5–12], are the most promising sources in terms of youthfulness [4]. MSCs can be retrieved from nearly 100% of UCs [13, 14] whereas UCB yields only 10%–63% [15–17]. Thus, UC-derived MSCs take advantage of their high availability and efficiency in MSC retrieval [13, 18]. 

MSCs are located within the abundant extracellular matrix of Wharton's jelly, where a number of growth factors exist. These growth factors include acid fibroblast growth factor, basic fibroblast growth factor (bFGF), transforming growth factor-*β*, insulin-like growth factor-I, platelet-derived growth factor, and epidermal growth factor [19]. bFGF is known as a stimulator of self-renewal, cell survival, and adhesion in undifferentiated human embryonic stem cells, as well as a regulator of differentiation in committed cells [20]. bFGF restores telomerase activity and maintains the self-renewal potential of endothelial cells [21]. In addition, bFGF acts as a local regulator of bone formation from osteoblast precursors. Conversely, osteoblasts produce bFGF and store it in the extracellular matrix as a bioactive form to regulate osteogenesis [22, 23]. Recently, transforming growth factor-*β*, platelet-derived growth factor, and FGF were reported as sufficient and essential factors for MSC differentiation [24].

The enzymatic digestion method, which destroys the extracellular meshwork, has been widely used to isolate MSCs from UCs, but the lengthy procedure of sequential enzymatic treatments results in low yields of only 1.0–2.4 × 10^5^ cells/cm cord [4, 25, 26] without any growth factors. To overcome the inefficiency of the enzymatic method, we adopted the explant culture method and compared the two methods for parameters such as characteristics of MSCs, the level of bFGF as a representative growth factor from UCs, and MSC gene expression profiles. 

## 2. Materials and Methods

### 2.1. Samples

UCs were obtained from newborns (38–41 weeks gestation) delivered from healthy mothers, without any perinatal problems. The Institutional Review Board of the Seoul National University Boramae Hospital approved the collection of UCs and UCB for research and cord blood banking. UCs were immediately transferred to the laboratory in PBS supplemented with 100 U/mL penicillin, 0.1 mg/mL streptomycin, and 0.25 mg/mL amphotericin B. UCs were washed twice with PBS to remove remnant blood. The net weight of each UC was measured. 

A total of 12 UCs were collected from term newborns with 38~42 gestational weeks without any evidence of congenital anomalies, and the average weight per length of UCs was 1.2 g/cm (range 0.7–1.75 g/cm).

### 2.2. Chemical and Reagents

Fetal bovine serum (FBS), Low-Glucose Dulbecco's Modified Eagle's Medium (L-DMEM), streptomycin, penicillin, amphotericin B, trypan blue, trypsin, and phosphate-buffered saline (PBS, pH 7.4) were purchased from Gibco BRL (Grand Island, NY, USA). CCK-8 was purchased from Dojindo Molecular Technologies, Inc. (Rockville, MA, USA). bFGF enzyme-linked immunoassay (ELISA) kits, osteocalcin antibody, and anti-rabbit IgG-horseradish peroxidase conjugated antibody were purchased from R&D Systems, Inc. (Minneapolis, MN, USA). TRI reagent, collagenase type II, glycerol-2-phosphate, dexamethasone, ascorbic-2-phosphate, sucrose, aprotinin, phenylmethylsulfonyl fluoride (PMSF), leupeptin, and goat serum were purchased from Sigma-Aldrich (St. Louis, MO, USA). All other chemicals utilized in this study were of the highest purity available from commercial sources. 

### 2.3. Explant Culture

After vessel removal, Wharton's jelly was dissected into small segments (diameter of 2-3 mm). The segments were cultured in 100 mm petri dishes with L-DMEM containing 10% FBS and antibiotics in a humidified 37°C, 5% CO_2_ incubator. The culture media were replaced twice a week. After 2 weeks, the tissue segments were removed from the cultures, with MSC being cultured one additional week to reach confluence. These MSCs were designated as the P0 population. 

### 2.4. Traditional Enzymatic Digestion

MSCs were isolated by enzymatic digestion from Wharton's jelly, as previously described [25]. Equivalent amounts of Wharton's jelly segments were digested with 0.1% collagenase type II for 30 min at 37°C. Washed cells were cultured in L-DMEM containing 10% FBS and antibiotics in 100 mm dish in a humidified 37°C, 5% CO_2_ incubator. After 3-week culture, we harvested the P0 cells. The medium were changed twice a week. 

### 2.5. Cell Viability

Detached cell suspensions were incubated for 3 min at room temperature with an equal volume of 0.4% (w/v) trypan blue solution. Viable MSCs were counted using a dual-chamber hemocytometer and a light microscope. 

### 2.6. Immunophenotyping

MSCs at P2 were washed in PBS containing 2% (v/v) FBS and incubated with the following antibodies conjugated with fluorescent probes on ice for 30 min in the dark: FITC-labeled anti-human-CD29, CD31, CD45, HLA-DR, and CD105 antibodies; PE-labeled anti-CD13, CD34, CD73, and HLA-ABC antibodies. Cells were analyzed using the FACS Aria System and FACSDivaTM Software (BD). 

### 2.7. bFGF ELISAs

Culture supernatant fluids were collected on culture days 3, 6, 9, 12, 14, 18, and 21 when their media were refreshed. Samples were stored at −70°C before measurement using Quantikine Human FGF basic ELISA kit in SpectraMax Plus 384 (Molecular Devices Corporation, CA, USA). Assays were performed in duplicate. 

### 2.8. Total RNA Isolation and RT-PCR Analysis

Extraction of total RNA was conducted using TRI reagent following the manufacturer's instructions. RNA samples were reverse transcribed using AMV reverse transcriptase and amplified by PCR with gene-specific primers using Taq polymerase, as per the manufacturer's instructions. The primer sequences used were as follows: Oct4 [27], 470 bp, 5′-gagaatttgttcctgcagtgc-3′, 5′-gttcccaattccttccttagtg-3′; Nanog [27], 470 bp, 5′-acctatgcctgtgatttgtgg-3′, 5′-aagagtagaggctggggtagg-3′; Nucleostamin [27], 747 bp, 5′-cagagatcctcttggttgcag-3′, 5′-aatgaggcacctgtccactc-3′; Sox2 [28] 130 bp, 5′-ggcagctacagcatgatgcaggagc-3′, 5′-ctggtcatggagttgtactgcagg-3′; GAPDH was used as an internal control. PCR products were electrophoresed on 1.5% agarose gels and stained with ethidium bromide. 

### 2.9. Characterization of Cultured Cells by Microarray

Total RNA from MSCs at P1 state was extracted using the mirVana miRNA isolation kit (Applied Biosystems/Ambion, Austin, TX), according to the manufacturer's protocols. The purity and integrity of the total RNA were assessed using the Bioanalyzer (Agilent, Santa Clara, CA). Probe synthesis from total RNAs, hybridization, detection, and scanning were performed following the manufacturer's protocols (GeneChip Whole Transcript Sense Target Labeling Assay Manual from Affymetrix). For the data analysis, the fluorescence intensity was processed and measured using a GeneChip scanner 3000 7G (Agilent, Santa Clara, CA). The raw intensity values were background-corrected, log_2_-transformed, and then quantile-normalized by applying robust multiarray averaging as a normalization process, which has been previously described [29]. The multivariate permutation test was used to identify genes with an expressional change confidence level of 95% and a false discovery rate <1%. The false discovery rate was based on the proportion of the genes that were defined to be differentially expressed but were actually false positives. The biological annotation of listed genes was analyzed by David bioinformatics resources [30]. 

### 2.10. Statistical Analysis

Statistical analysis was performed using Wilcoxon signed rank test using the MedCalc software (version 12.0, Broekstraat, Belgium), and *P* values < 0.05 were considered statistically significant. 

## 3. Results

### 3.1. bFGF during Explant Culture of Wharton's Jelly

**Figure 1:**
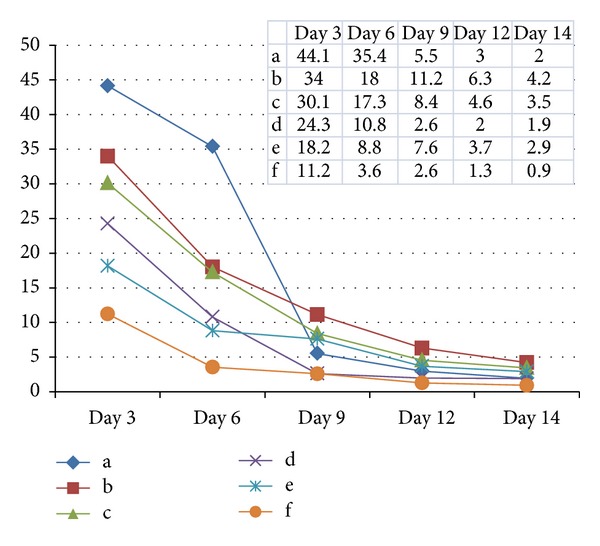
The levels of bFGF in supernatant media according to culture days. The letters (a~f) indicated each UC. And the levels were expressed ng per gram of Wharton's jelly tissue. The bFGF levels were highest on day 3 with 27.0 ± 11.7 (m ± SD) ng/g and declined afterwards, resulting in total amount of 55.0 ± 25.6 (m ± SD) ng/g.

The level of bFGF released during explant culture was highest of 27.0 ± 11.7 (m±SD) ng/g of Wharton's jelly at day 3, then declined to the level of 2.6 ± 1.2 at day 14 (Figure 1), and the differences between the dates were significant (*P* < 0.05). The sum of the bFGF was 55.0 ± 25.6 ng/g for the whole 14 days. 

Although the small sample number prevented drawing any differences between the two methods in the level of bFGF, explant culture method showed higher levels especially in earlier culture date (Table 1). 

### 3.2. Cell Number and Viability of MSC

Explant-derived MSCs generated 2.8 times greater number of cells than enzymatic digestion-derived MSCs at P0 (*n* = 7,  *P* = 0.01, 4.89 × 10^5^ ± 3.2 × 10^5^ cells and 1.75 × 10^5^ ± 2.2 × 10^5^ cells/g Wharton's jelly, resp.) (Table 2). 

### 3.3. Characteristics of Explant Derived MSC

**Figure 2:**
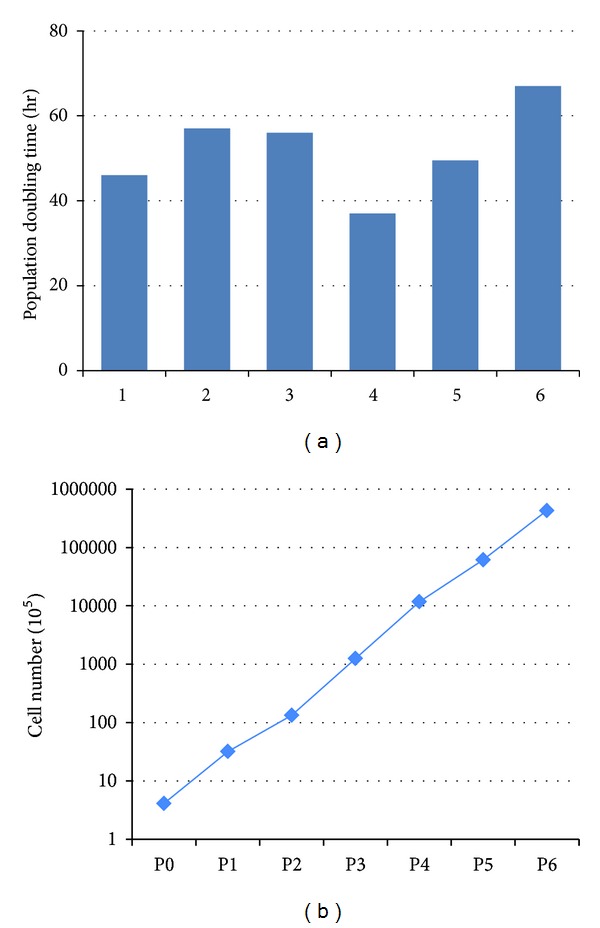
Population doubling time (hrs) of Wharton's jelly-derived MSCs during passages 1–6. The initial cells (4 × 10^5^ cells) expanded to 4.2 × 10^10^ cells in 23.3 days (passage 6).

Until day 23, passage 6, we could get 1 × 10^5^ cells from a single explant-cultured MSC with population doubling time < 3 days (Figure 2). 

**Figure 3:**
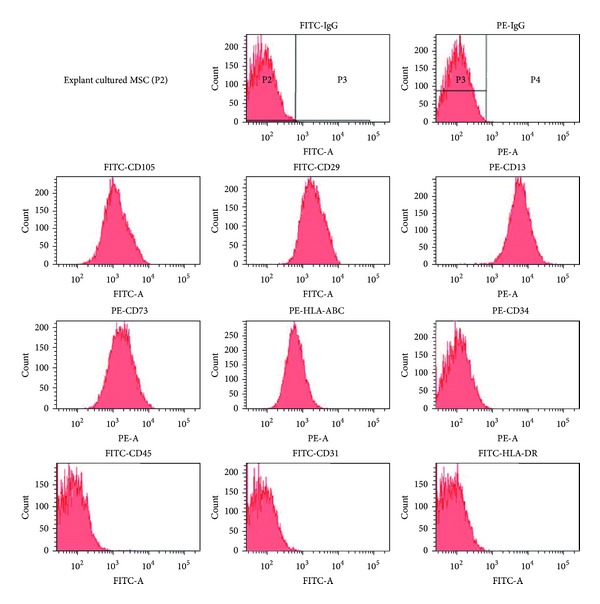
Cell surface marker analysis of P2 explant cultured MSCs. MSCs expressed CD13, CD29, CD73, CD105, and HLA-ABC but did not express CD34, CD31, CD45, and HLA-DR.

**Figure 4:**
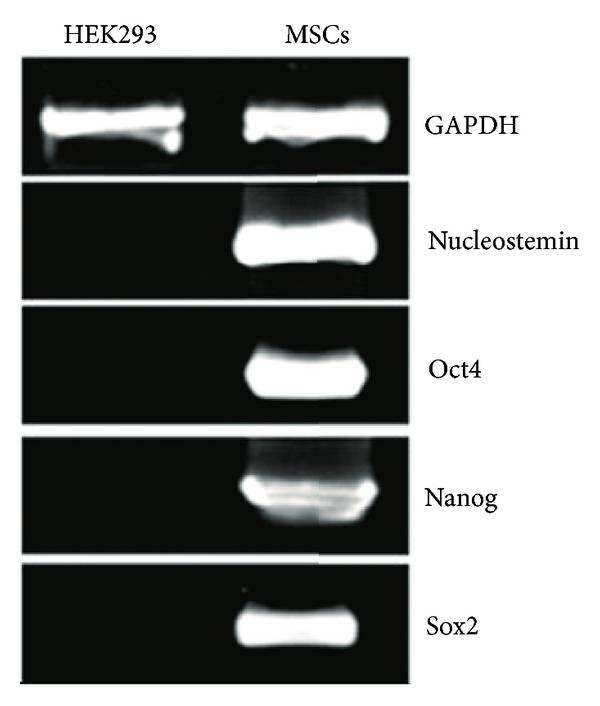
Expression of stem cell-related genes and growth kinetics of Wharton's jelly-derived MSC. MSCs expressed Oct4, Nanog, Sox2, and Nucleostemin mRNAs. GAPDH was used as an internal control, and HEK293 cells were used for negative control.

Cell surface markers showed positive for CD105, CD29, CD13, CD73, and HLA class I and negative for CD34, CD45, CD31, and HLA class II (Figure 3), which met the cell surface criteria for MSC [31]. The MSC isolated from Wharton's jelly using explant culture showed stem cell characteristic with expression of OCT4, Sox2, Nanog, and Nucleostemin genes (Figure 4). 

### 3.4. Comparison of mRNA Expression between the Methods

**Figure 5:**
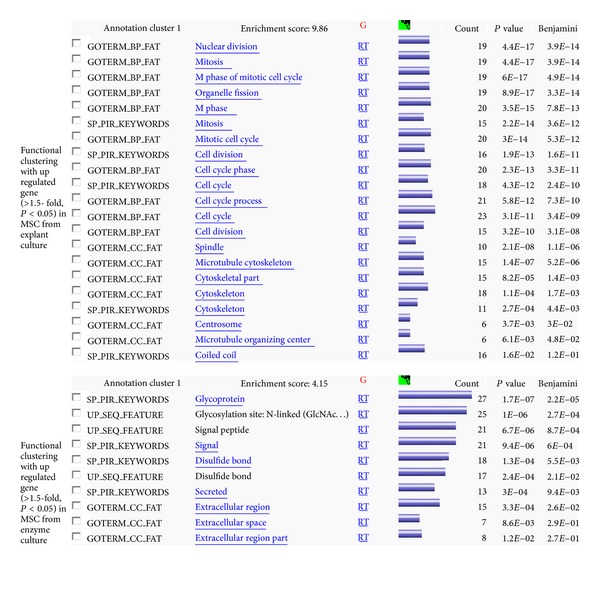
Gene clusters deduced from the differences of mRNA expression between explant-derived and enzymatic digestion-derived MSCs.

Microarray analysis represented that 1,398 genes among 21,331 annotated genes were determined as significantly up- or downregulated (*P* < 0.05) in explant-derived MSCs. There were 150 genes which showed >1.5 fold difference of expression. The upregulated genes were clustered into similar gene ontology (GO) groups genes related to mitosis, M phase of mitotic cell cycle, M phase, mitotic cell cycle, cell cycle phase, cell cycle process, and related cell cycle (Figure 5). 

## 4. Discussion

Previous studies have described detailed procedures for the isolation of MSCs from human cord stroma and Wharton's jelly [4, 25, 26, 32, 33]. In those procedures, various protein-destroying enzymes were used to isolate the cells from the extracellular matrix within the primary culture. The explant culture procedure in this study prevented possible cellular damage induced by such enzymes and resulted in increased viability and higher yield of MSCs, and this result is in accordance with Ishige's report [34]. 

In terms of the yield and viability of MSCs, explant culture was superior to enzymatic method. Until day 23, passage 6, we could get 1 × 10^5^ cells from single cell with population doubling time of less than 3 days. After converting previously reported yields from cells/cm to cells/g of UC using the mean g weight per cm-length from our study (1.21 ± 0.4 g/cm, *n* = 12), this explant culture method yielded 3.9~87 times more cells per gram of UC [4, 25, 26]. The array results support this higher cellular yield by higher expression of mitosis and cell cycle related genes in explant-derived MSCs. 

There are many clinical trials using MSCs such as multiple injections of BM-derived MSC for overcoming acute GVHD [35]. One of the key factors for successful cellular therapy using MSC is the amount of cells available for transplantation to provide a therapeutic effect. The cells from early passages are preferred for cellular therapy [36]. In terms of these facts, larger number of MSCs in early passages is required from a single source and single donor. 

The MSCs isolated from Wharton's jelly using explant culture expressed the OCT4, Sox2, Nanog, and Nucleostemin genes, which are the characteristic of stem cells. The Sox2 gene is expressed in Wharton's jelly-derived MSC but not in umbilical vein-derived MSC, which suggests that Wharton's jelly-derived MSC have strong stem cell characteristics [27]. This supports the superiority of MSCs from Wharton's jelly among various sources of MSCs. 

The thickness and composition of UCs varied between individuals and their gestational week (GW) of pregnancy [37]. Although we tried to reduce the individual variations by estimation based on UC weights, rather than lengths, we observed large differences among levels of bFGF at the earlier time points (Figure 1) which remains to be further investigated. 

The declining of bFGF levels in culture with time was evident (Figure 2). In terms of the origin of the cytokine, bFGF was released from the remaining mass of tissue in the primary culture plate. After removal of tissue, the main source of cytokine is MSC, from which relatively small amount of bFGF was produced in vitro. Likewise, we can deduce that the enzyme digestion-derived MSCs had ragged tissue remnant around the cells and the supernatant has some cytokine released from the tissue. 

bFGFs regulate the growth and proliferation of many different cells, including MSCs, and affect osteogenic differentiation [24]. When 5 ng/mLlbFGF was added to culturing bone-marrow-derived MSCs, the percentage of mineralized area was maximized at 89% [34], and 5 ng/mL FGF increased proliferation [38]. The amount of bFGF naturally released in explant culture was greater than that produced by the tissue homogeniser-ultrasonication procedure (53.2 ng/g versus 20.51 ng/g) [19]. 

To the best of our knowledge, this is the first report to measure the level of natural bFGF released from human UCs. Some reports characterized the explant-derived MSCs using full MSC markers on cells and the gene expression presented by both protein and mRNA levels [13]. However, they did not measure either the yield of MSCs or any growth factors that could justify their procedure. 

Wharton's jelly is one of the most valuable sources of MSCs that can be obtained without any ethical issues. This is the first report to verify the advantages of explant culture of Wharton's jelly for acquiring both MSCs and natural cytokine represented as bFGF.

## Figures and Tables

**Table 1:** The levels of bFGF released to the supernatants of explant and enzymatic culture method (*n* = 3).

	Culture with tissue					After tissue removal	
	Day 3	Day 6	Day 7	Day 12	Day 14	Day 18	Day 21
Explant culture (mean ± SD, ng/g of Wharton's jelly)	41.8 ± 7.0	23.6 ± 10.3	8.4 ± 2.8	4.6 ± 1.7	3.2 ± 1.2	2.1 ± 0.8	2.0 ± 0.9
Enzyme digestion (mean ± SD, ng/g of Wharton's jelly)	23.5 ± 6.2	8.4 ± 8.3	3.9 ± 3.2	2.2 ± 0.4	2.1 ± 0.3	2.7 ± 1.5	3.1 ± 1.9

**Table 2:** Comparison of quality of MSCs (P0) derived from explant culture and enzyme-treated culture.

	Explant-derived MSCs	Enzyme digestion-derived MSCs	
Cell number (*n* = 7)	4.89 × 10^5^/g (±3.2)	1.75 × 10^5^/g (±2.2)	*P* = 0.01
Viability (*n* = 5)	96.1% (±1.2)	85.1% (±1.4)	*P* < 0.001

## References

[1] Caplan AI, Dennis JE (2006). Mesenchymal stem cells as trophic mediators. *Journal of Cellular Biochemistry*.

[2] Li G, Zhang X-A, Wang H (2009). Comparative proteomic analysis of mesenchymal stem cells derived from human bone marrow, umbilical cord, and placenta: implication in the migration. *Proteomics*.

[3] Chao KC, Chao KF, Fu YS, Liu SH (2008). Islet-like clusters derived from mesenchymal stem cells in Wharton's jelly of the human umbilical cord for transplantation to control type 1 diabetes. *PLoS ONE*.

[4] Weiss ML, Medicetty S, Bledsoe AR (2006). Human umbilical cord matrix stem cells: preliminary characterization and effect of transplantation in a rodent model of Parkinson's disease. *Stem Cells*.

[5] Alviano F, Fossati V, Marchionni C (2007). Term amniotic membrane is a high throughput source for multipotent mesenchymal stem cells with the ability to differentiate into endothelial cells in vitro. *BMC Developmental Biology*.

[6] Hu Y, Liao L, Wang Q (2003). Isolation and identification of mesenchymal stem cells from human fetal pancreas. *Journal of Laboratory and Clinical Medicine*.

[7] In 't Anker PS, Noort WA, Scherjon SA (2003). Mesenchymal stem cells in human second-trimester bone marrow, liver, lung, and spleen exhibit a similar immunophenotype but a heterogeneous multilineage differentiation potential. *Haematologica*.

[8] Miao Z, Jin J, Chen L (2006). Isolation of mesenchymal stem cells from human placenta: comparison with human bone marrow mesenchymal stem cells. *Cell Biology International*.

[9] Nomura T, Ashihara E, Tateishi K (2007). Therapeutic potential of stem/progenitor cells in human skeletal muscle for cardiovascular regeneration. *Current Stem Cell Research and Therapy*.

[10] Peng L, Jia Z, Yin X (2008). Comparative analysis of mesenchymal stem cells from bone marrow, cartilage, and adipose tissue. *Stem Cells and Development*.

[11] Perry BC, Zhou D, Wu X (2008). Collection, cryopreservation, and characterization of human dental pulp-derived mesenchymal stem cells for banking and clinical use. *Tissue Engineering—Part C*.

[12] Zvaifler NJ, Marinova-Mutafchieva L, Adams G (2000). Mesenchymal precursor cells in the blood of normal individuals. *Arthritis Research*.

[13] La Rocca G, Anzalone R, Corrao S (2009). Isolation and characterization of Oct-4^+^/HLA-G^+^ mesenchymal stem cells from human umbilical cord matrix: differentiation potential and detection of new markers. *Histochemistry and Cell Biology*.

[14] Secco M, Zucconi E, Vieira NM (2008). Mesenchymal stem cells from umbilical cord: do not discard the cord!. *Neuromuscular Disorders*.

[15] Bieback K, Kern S, Klüter H, Eichler H (2004). Critical parameters for the isolation of mesenchymal stem cells from umbilical cord blood. *Stem Cells*.

[16] Erices A, Conget P, Minguell JJ (2000). Mesenchymal progenitor cells in human umbilical cord blood. *British Journal of Haematology*.

[17] Kern S, Eichler H, Stoeve J, Klüter H, Bieback K (2006). Comparative analysis of mesenchymal stem cells from bone marrow, umbilical cord blood, or adipose tissue. *Stem Cells*.

[18] Weiss ML, Anderson C, Medicetty S (2008). Immune properties of human umbilical cord Wharton's jelly-derived cells. *Stem Cells*.

[19] Sobolewski K, Małkowski A, Bańkowski E, Jaworski S (2005). Wharton's jelly as a reservoir of peptide growth factors. *Placenta*.

[20] Eiselleova L, Matulka K, Kriz V (2009). A complex role for FGF-2 in self-renewal, survival, and adhesion of human embryonic stem cells. *Stem Cells*.

[21] Kurz DJ, Hong Y, Trivier E (2003). Fibroblast growth factor-2, but not vascular endothelial growth factor, upregulates telomerase activity in human endothelial cells. *Arteriosclerosis, Thrombosis, and Vascular Biology*.

[22] Montero A, Okada Y, Tomita M (2000). Disruption of the fibroblast growth factor-2 gene results in decreased bone mass and bone formation. *The Journal of Clinical Investigation*.

[23] Xiao G, Jiang D, Gopalakrishnan R, Franceschi RT (2002). Fibroblast growth factor 2 induction of the osteocalcin gene requires MAPK activity and phosphorylation of the osteoblast transcription factor, Cbfa1/Runx2. *The Journal of Biological Chemistry*.

[24] Ng F, Boucher S, Koh S (2008). PDGF, tgf-2, and FGF signaling is important for differentiation and growth of mesenchymal stem cells (mscs): transcriptional profiling can identify markers and signaling pathways important in differentiation of MSCs into adipogenic, chondrogenic, and osteogenic lineages. *Blood*.

[25] Karahuseyinoglu S, Cinar O, Kilic E (2007). Biology of stem cells in human umbilical cord stroma: in situ and in vitro surveys. *Stem Cells*.

[26] Seshareddy K, Troyer D, Weiss ML (2008). Method to isolate mesenchymal-like cells from Wharton's Jelly of umbilical cord. *Methods in Cell Biology*.

[27] Kermani AJ, Fathi F, Mowla SJ (2008). Characterization and genetic manipulation of human umbilical cord vein mesenchymal stem cells: potential application in cell-based gene therapy. *Rejuvenation Research*.

[28] Klassen H, Ziaeian B, Kirov II, Young MJ, Schwartz PH (2004). Isolation of retinal progenitor cells from post-mortem human tissue and comparison with autologous brain progenitors. *Journal of Neuroscience Research*.

[29] Park W-Y, Hwang C-I, Im C-N (2002). Identification of radiation-specific responses from gene expression profile. *Oncogene*.

[30] Huang DW, Sherman BT, Lempicki RA (2009). Systematic and integrative analysis of large gene lists using DAVID bioinformatics resources. *Nature Protocols*.

[31] Dominici M, Le Blanc K, Mueller I (2006). Minimal criteria for defining multipotent mesenchymal stromal cells. The International Society for Cellular Therapy position statement. *Cytotherapy*.

[32] Lu L-L, Liu Y-J, Yang S-G (2006). Isolation and characterization of human umbilical cord mesenchymal stem cells with hematopoiesis-supportive function and other potentials. *Haematologica*.

[33] Sarugaser R, Lickorish D, Baksh D, Hosseini MM, Davies JE (2005). Human umbilical cord perivascular (HUCPV) cells: a source of mesenchymal progenitors. *Stem Cells*.

[34] Sotiropoulou PA, Perez SA, Salagianni M, Baxevanis CN, Papamichail M (2006). Characterization of the optimal culture conditions for clinical scale production of human mesenchymal stem cells. *Stem Cells*.

[35] Kebriaei P, Isola L, Bahceci E (2009). Adult human mesenchymal stem cells added to corticosteroid therapy for the treatment of acute graft-versus-host disease. *Biology of Blood and Marrow Transplantation*.

[36] Halfon S, Abramov N, Grinblat B, Ginis I (2011). Markers distinguishing mesenchymal stem cells from fibroblasts are downregulated with passaging. *Stem Cells and Development*.

[37] Yinze D, Ma Q, Cui F, Zhong Y (2009). Human umbilical cord mesenchymal stem cells: osteogenesis *vivo* as seed cells for bone tissue engineering. *Journal of Biomedical Materials Research—Part A*.

[38] Bianchi G, Banfi A, Mastrogiacomo M (2003). Ex vivo enrichment of mesenchymal cell progenitors by fibroblast growth factor 2. *Experimental Cell Research*.

